# Le gliome du tronc cérébral : cause rare de vertige central de l’adulte

**DOI:** 10.11604/pamj.2016.25.135.10669

**Published:** 2016-11-04

**Authors:** Senda Turki, Ali Mardassi, Safa Nefzaoui, Amani Hachicha, Sofiène Ben Rhouma

**Affiliations:** 1 Service ORL, Hôpital FSI,Faculté de Médecine de Tunis, Université Tunis El Manar

**Keywords:** Gliome, tronc cérébral, vertige, vidéonystagmographie, radiothérapie, Glioma, brainstem, vertigo, videonystagmography, radiation therapy

## Abstract

Contrairement à ceux de l'enfant, les gliomes du tronc cérébral de l'adulte sont rares et représentent un groupe hétérogène de tumeurs, souvent de bas grade et de meilleur pronostic. Ces tumeurs représentent une cause rare de vertige central chez l'adulte. Le traitement des gliomes diffus repose sur la radiothérapie. A travers un cas de gliome du tronc cérébral de bas grade chez une femme de 35 ans révélé par des troubles de l'équilibre, ce travail développera les différents aspects cliniques, paracliniques et radiologiques de cette affection, ainsi que les moyens thérapeutiques et les modalités évolutives.

## Abstract (en)

Unlike children, brainstem gliomas in adults are rare and represent a heterogeneous group of tumors: often low grade tumors having a better prognosis. These tumors are a rare cause of central vertigo in adults. The treatment of diffuse gliomas is based on radiation therapy. We here report the case of a 35-year old female patient with low grade brainstem glioma revealed by balance disorders, in order to highlight the different clinical, paraclinical and radiological aspects of this disease as well as the therapeutic tools and the progressive methods.

## Introduction

Les gliomes du tronc cérébral sont rares chez l'adulte (moins de 2% de l'ensemble des gliomes) [1]. Il s'agit d'un groupe hétérogène de tumeurs, classées en trois grandes catégories selon leur présentation clinique et histo-radiologique. Le type infiltrant diffus de bas grade prédomine chez l'adulte [2]. Ces tumeurs sont connues par leurs manifestations cliniques souvent insidieuses et leur pronostic plus favorable que celles survenant chez l'enfant [1].

## Patient et observation

**Figure 1:**
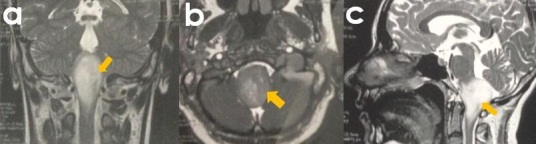
IRM cérébrale montrant un gliome de bas grade du tronc cérébral : a): coupe frontale en T1 injectée; b): coupe axiale en T1 injectée; c): coupe sagittale en T2

Madame X, âgée de 35 ans et sans antécédents pathologiques notables, s'est présenté à nos consultations pour troubles de l'équilibre évoluant depuis 4 mois, à type de bascule à la marche, plus fréquente du côté gauche, responsables d'une agoraphobie. La patiente a rapporté également la sensation de chute et d'enfoncement dans son lit en décubitus latéral gauche, ainsi qu'un épisode de « Drop-Attack ». Ces symptômes étaient accompagnés de paresthésies de l'hémicorps droit et d'une hypoacousie gauche avec des acouphènes. L'examen vestibulaire a retrouvé une patiente instable à l'épreuve de Romberg et un Gaze nystagmus de type torsionnel. La manœuvre de Dix Hallpike a révélé un nystagmus inférieur durable, non inversé au redressement mais devenant horizontal gauche. Le Head Schaking Test a mis en évidence un nystagmus gauche. L'examen neurologique n'a retrouvé aucun déficit notable. A l'otoscopie, il existait une perforation tympanique sèche du côté gauche avec un test de Weber latéralisé à gauche à l'acoumétrie. La vidéonystagmographie (VNG) a mis en évidence une prépondérance calorique droite sans hypovalence, ainsi qu'un nystagmus positionnel atypique de type central. Devant ce contexte évocateur d'une cause centrale, la patiente a bénéficié d'une imagerie par résonance magnétique cérébrale (IRM) qui a objectivé un gliome du tronc cérébral, infiltrant, diffus de bas grade (Figure 1). La patiente a été traitée par radiothérapie de type conformationnelle (30 séances de 2Gy chacune). Dans les suites, elle a présenté une instabilité permanente avec des oscillopsies dans le regard latéral, sans vertige. Une rééducation vestibulaire orientée et régulière a permis une amélioration fonctionnelle significative, avec autonomie de déplacement, au bout de trois mois.

## Discussion

L'originalité de ce cas clinique réside dans la révélation d'un gliome du tronc cérébral par une manifestation cochléo-vestibulaire prédominante. En effet, en dehors des processus expansifs de l'angle ponto-cérébelleux et préférentiellement du neurinome de l'acoustique, il est rare qu'une affection primitive du tronc cérébral ait une telle présentation clinique. Les gliomes du tronc cérébral représentent un groupe hétérogène de tumeurs, classées chez l'adulte en quatre grandes catégories : les gliomes intrinsèques diffus de bas grade (représentant la moitié des cas), les gliomes malins du tronc (survenant dans un tiers des cas), les tumeurs exophytiques et les gliomes focaux tectaux (10 % des cas) [1]. Les gliomes intrinsèques de bas grade, de loin les plus fréquents, surviennent en général chez des patients jeunes (âge médian de 34 ans lors du diagnostic) [1–3]. La durée moyenne des symptômes avant le diagnostic est longue (plus de 3 mois), ce qui reflète le caractère lentement évolutif de ces tumeurs [3]. La présentation clinique associe une atteinte des nerfs crâniens, une ataxie, des troubles de la marche et des signes déficitaires moteurs par atteinte des voies longues [3, 4]. Des céphalées et des signes d'hypertension intracrânienne peuvent être observés dans les formes extensives. Notre patiente ne présentant pas le cortège neurologique classique, l'examen vestibulaire détaillé révélant des signes centraux nous a incité à demander une imagerie. La maladie peut se manifester aussi par une paralysie faciale ou un hémispasme de la face. D'autres symptômes comme des troubles respiratoires d'origine neurologique, une pseudo-myasthénie ou un syndrome parkinsonien peuvent être retrouvés [5, 6]. L'examen clinique doit rechercher des signes cérébelleux ou une atteinte des nerfs crâniens (notamment des nerfs V, VI, VII et VIII), qui orientent vers une cause centrale. L'IRM montre un aspect d'élargissement diffus du tronc cérébral en hypersignal T2 et en hyposignal T1 sans prise de contraste à l'injection de gadolinium [6, 7]. Plusieurs études ont montré une concordance du diagnostic établi par l'IRM et l'examen anatomopathologique dans plus de 95 % des cas. Ainsi, en cas d'image caractéristique d'infiltration diffuse sans prise de contraste à l'IRM, la biopsie diagnostique n'est pas requise vue la spécificité de l'IRM et le risque de morbidité de ce geste [6]. En dehors de cette image caractéristique, la biopsie permet d'éliminer les lésions inflammatoires et infectieuses [6].

D'autres auteurs prônent l'indication systématique d'une biopsie stéréotaxique afin d'établir un diagnostic de certitude. Il peut en effet s'agir de métastases, lymphome, cavernomes, anomalies vasculaires ou de lésions inflammatoires et démyélinisantes [1, 4]. La radiothérapie est le traitement de choix du gliome de bas grade du tronc cérébral de l'adulte [3, 7]. En effet, ces tumeurs paraissent beaucoup plus sensibles à la radiothérapie chez l'adulte que chez l'enfant [3]. La dose moyenne recommandée est de 50- 55 Gy avec un fractionnement de 1,8 à 2 Gy [1]. L'amélioration clinique s'observe chez la plupart des patients, néanmoins, il existe une discordance entre la réponse radiologique et clinique. Des glucocorticoïdes sont généralement associés, afin de réduire l'œdème et d'améliorer les symptômes en rapport avec la compression des voies longues [6]. La résection chirurgicale n'a pas de place dans la prise en charge de ces tumeurs, souvent infiltrantes et inaccessibles. Par contre, une dérivation du liquide céphalorachidien peut être indiquée en cas de symptômes importants [3]. La chimiothérapie n'a pas montré son efficacité. Son utilisation concomitante à la radiothérapie peut être discutée, avec une attention particulière à la détection des signes de myélotoxicité [1, 3]. Contrairement à l'enfant chez qui ces tumeurs sont de mauvais pronostic avec une médiane de survie inférieure à un an, chez l'adulte, l'évolution du gliome de bas grade est lente et progressive avec une médiane de survie de l'ordre de sept ans [3, 5]. Toutefois, ces tumeurs peuvent revêtir un profil évolutif défavorable, par une croissance plus rapide ou une transformation vers un gliome de haut grade caractérisée par l'apparition d'une prise de contraste de la masse à l'IRM.

## Conclusion

Les gliomes du tronc cérébral chez l'adulte représentent un groupe de tumeurs hétérogènes et sont rarement à l'origine d'un tableau cochléo-vestibulaire inaugural. Ils sont caractérisés par une évolution insidieuse et un pronostic plutôt favorable. Une meilleure connaissance des caractéristiques moléculaires de ces tumeurs est requise afin de développer d'autres moyens thérapeutiques spécifiques.
